# Peanut Allergy in Children—Is Prevention Better than Cure?

**DOI:** 10.3390/nu16193237

**Published:** 2024-09-25

**Authors:** Alicja Krejner-Bienias, Klementyna Łyżwa, Anna Krupa-Łaska, Joanna Zielińska, Marek Kulus, Katarzyna Grzela

**Affiliations:** Department of Pediatric Pneumonology and Allergy, Medical University of Warsaw, 02-091 Warsaw, Poland

**Keywords:** children, food allergy, oral immunotherapy, peanut

## Abstract

Peanut allergy, one of the most frequently occurring allergies, usually starts in childhood and rarely subsides—often persisting throughout adult life. Accidental exposure to peanuts can often result in adverse reactions ranging from mild to life-threatening, such as anaphylactic shock. Historically, food avoidance and the use of rescue drugs have remained a fundamental management mechanism for dealing with food allergy. However, prevention of adverse reactions to food allergy is playing an increasing role. This is possible through the early introduction of peanuts into the diet, especially in infants at risk of this allergy. In recent years, specific immunotherapy has been used to develop desensitisation and, in some patients, tolerance—defined as a persistent state of clinical non-reactivity to the allergen after therapy is finished. The aim of this article is to summarise the current state of knowledge on the prevention and treatment of peanut allergy, with a focus on clinical trials, current guidelines, and recent experimental studies. This review may be particularly useful for paediatricians and general practitioners.

## 1. Introduction

Food allergy (FA) is a significant and increasing clinical and social problem, especially in children. FA affects approximately 3% of children in Europe [1].

Food sensitisation (FS), alongside other features expressed as the presence of allergen-specific antibodies, is a prerequisite for the development of FA. The presence of allergen sensitisation can be determined by in vivo and in vitro tests. In vitro tests can be used to identify total and specific IgE in peripheral blood [2,3], whereas in vivo skin prick tests determine the functional role of these antibodies in type I immediate allergic reactions [4,5]. FA is diagnosed when sensitisation is accompanied by clinical symptoms after exposure to the allergen.

Peanut (*Arachis hypogaea*) belongs to the most frequent food allergens [6]. In most cases, peanut allergy starts in childhood and usually persists throughout adult life [7]. It is estimated that only 20% of patients develop tolerance [8]. So far, scientists have identified 17 peanut proteins that are registered as “Ara h” molecules by the World Health Organisation (WHO) and the International Union of Immunological Societies Allergen Nomenclature Sub-Committee (WHO/IUIS) [9]. They belong to a group of heat- and digestion-resistant seed storage proteins and can cause life-threatening anaphylactic reactions [10]. 

To prevent adverse reactions, people with IgE-mediated FA should strictly avoid allergen-containing food. Unintentional ingestion can cause adverse reactions, ranging from mild (e.g., itching and hives) to life-threatening, such as anaphylactic shock. Therefore, patients and their caregivers should be trained in the administration of epinephrine with an autoinjector in the event of anaphylaxis [11]. 

Elimination diet, social isolation, fear of accidental allergen ingestion, and the need to use medication in the event of an allergic reaction all have detrimental effects on the quality of life of patients with FA and their families [12].

So far, food avoidance, waiting for natural tolerance achievement, and rescue drug use have remained fundamental management strategies in FA.

Currently, an increasing role is being given to the possibility of developing food tolerance. This is possible through the early introduction of allergenic foods into patients’ diets, especially in infants at risk of FA [13].

In recent years, specific immunotherapy has also played an important part in the management of food allergy (mainly oral immunotherapy, OIT). This method allows patients to develop desensitisation and, in some cases, tolerance, which can be defined as a persistent state of clinical non-reactivity to the allergen after therapy is finished. The European Academy of Allergy and Clinical Immunology (EAACI) recommends food oral immunotherapy for the treatment of peanut, milk, and egg allergy [14].

The aim of this paper is to summarise the current understanding of peanut allergy prevention and treatment. We focused on the results of existing clinical trials and current guidelines. 

## 2. Allergen Avoidance

Currently, peanut OIT is only available to a limited number of patients with FA. Thus, a restrictive peanut-free diet is a must for most of them.

Notwithstanding dietary avoidance, reactions after accidental exposure to peanuts occur frequently with an annual incidence rate of 12–14% [15,16]. Since strict avoidance of peanut-containing food is necessary for allergic people to prevent the occurrence of severe allergic reactions, manufacturers must label such products following the Codex Alimentarius [17].

The Codex Alimentarius, or “Food Code”, is a list of standards and guidelines constructed to preserve the health of consumers and ensure fair practices in the food trade. Despite these guidelines, labelling regulations vary in different countries [18].

The European Union’s Food Information Regulation No. 1169/2011 has defined 14 major food allergens, including peanuts. Labelling of foods with these allergens is mandatory in the EU [19]. 

Nevertheless, patients with food allergy can still be unintentionally exposed to peanuts because of food contamination from processing lines that share peanut products. Precautionary allergen labelling (PAL), such as “may contain peanuts” or “may contain traces of peanuts”, is used voluntarily by food manufacturers [20]. 

However, PAL is used inconsistently, and it does not always demonstrate the actual risk to consumers. 

Underestimating the content of peanuts may pose a threat to life. On the other hand, overestimating peanut contamination may cause unnecessary anxiety in people with allergy [21,22]. Thus, reliable information about the content of “Ara h” allergens would be relevant both for patients and their doctors. 

The need to follow a restrictive diet leads to numerous difficulties: social isolation, resignation from school and holiday trips, and an inability to attend restaurants or use public transport [21]. 

Food allergies can also affect the activities of entire families. Bollinger et al. have shown that, among surveyed caregivers, more than 60% reported that FA had a significant impact on meal preparation. A total of 49% or more expressed that FA affected family and social activities. Parents of children with FA have also been found to be more mindful and vigilant of their surroundings in case of potential allergic reactions [23]. The social consequences of having FA have also been shown to considerably impact the school environment. In a study by Fong and colleagues, 32% of children reported being subjected to bullying at least once on account of their food allergy [24].

## 3. Allergy Prevention

FA has been shown to lead to morbidity and psychosocial burden (including the risk of nutritional deficiencies and life-threatening anaphylaxis) and have a negative impact on quality of life [12,25]. The effects of an elimination diet, vitamin supplementation, and probiotic and prebiotic addition were evaluated. Neither dietary management nor using emollients prevented the occurrence of FA in children [26]. 

So far, the early introduction of allergenic foods in infancy has been shown to be one of the most promising strategies to decrease the risk of FA, especially in high-risk families. At the beginning of the 20th century, most scientific societies, including the American Academy of Pediatrics, recommended a delay to the introduction of potentially allergenic foods into patients’ diets until 3 years of age [27]. The premise of this theory was that the lack of exposure to allergenic foods during early infancy would prevent the later development of FA [28]. Over the past decade, after the publication of the first meta-analysis on the early introduction of peanuts into the diet of children with allergic diseases in the *New England Journal of Medicine* (the LEAP study), the allergy prevention guidelines have changed. Du Toit et al. showed a statistically significant reduction in peanut allergy in the group introduced to peanuts early when compared with the avoidance group. The LEAP study included 640 infants at high risk for peanut allergy (defined as those with severe eczema and/or egg allergy) aged between 4 and 11 months. The early introduction of peanuts was proven to decrease the prevalence of IgE-mediated peanut allergy by over 80% at 5 years of age [13].

The EAT study analysed the impact of the early introduction of allergenic foods (including peanuts) into the diet of infants being breastfed as compared to the general population without predefined risk criteria of allergy. The authors proved the beneficial effect of early food introduction. The prevalence of FA was considerably lower in the early introduction group (3 months of age) than in the standard introduction group (approximately 6 months of age), according to the per-protocol analysis [29]. Although the results of the EAT study did not show as spectacular of a reduction in the development of food allergy as the LEAP study, analysis of both studies indicates that the incidence of FA can be markedly reduced by introducing allergenic foods into the diet at an early age.

It is likely that the efficacy of introducing peanuts into diets early depends on the risk profile of allergy development. The known risk factors of peanut allergy include family history of allergy, genetic factors (e.g., filaggrin mutation), early onset and severe eczema, egg allergy, cutaneous exposure to food allergens, and delayed peanut introduction [30,31,32,33,34,35].

According to the dual exposure hypothesis, a disrupted skin barrier may predispose infants with early-onset eczema to peanut sensitisation. Thus, previous consumption of peanut proteins could prevent sensitisation [36]. Analysis of clinical trials has shown that infants with moderate–severe eczema and/or early-onset eczema benefit most from the early introduction of peanuts (if tolerated) into the diet.

The results of the LEAP study have completely changed the rules of expanding children’s diets with peanuts. The guidelines of all major societies consistently recommend introducing peanuts into diets quickly (between 4 and 12 months of age), including in infants at high risk of peanut allergy and in geographies with a high prevalence of it [37,38,39,40,41].

While the authors of the above recommendations agree that early introduction of peanuts may be beneficial in reducing the risk of peanut allergy, there is some disagreement about whether allergy tests should be performed in high-risk infants before their first peanut consumption.

The National Institute of Allergy and Infectious Diseases guidelines strongly recommend an evaluation with a peanut skin prick test (SPT) and/or a peanut-specific immunoglobulin E (sIgE) measurement prior to peanut introduction in infants at high risk of allergy (severe eczema and/or egg allergy and LEAP study entry criteria) [42]. Conversely, the Australasian Society of Clinical Immunology and Allergy recommends the introduction of peanuts into infants’ diets regardless of risk factors and without screening [41]. Similar recommendations were published by the Canadian Paediatric Society (CPS). They encourage the early (at 4–6 months) introduction of allergenic foods to high- and low-risk infants in a safe and age-appropriate manner in a home setting. The CPS emphasises the need for regular ingestion of allergenic foods (if introduced and tolerated) to maintain tolerance [43]. 

Most guidelines do not suggest obligatory allergic screening of infants. Testing all infants at high risk of peanut allergy (e.g., with eczema) is costly and difficult. Moreover, peanut allergy occurs in infants without commonly used risk criteria. In the vast majority of patients, a negative skin prick test helps to exclude immediate-type allergy, whereas a positive skin test confirms only sensitisation and does not mean that the infant has FA. Additionally, up to 50% of positive tests in this age group are false positives [44]. Hence, allergic testing should only be considered in those children whose parents are concerned about administering peanuts at home or should be performed if there is a possibility of quick peanut introduction under medical supervision. Tests are not recommended in children who have already tolerated any portions of food [39].

There are some challenges associated with the broad implementation of guidelines on the early introduction of peanuts in patients’ diets. One of the barriers to overcome may be difficulty in changing existing habits, both among physicians and parents. Greenhawt et al. investigated the level of support for early peanut introduction among new and expectant caregivers of infants at risk of peanut allergy. Although 54% of them felt that the age at which certain foods were introduced into the diet was important in the development of FA, only 31% showed a willingness to introduce peanuts before or around 6 months of age, with 40% expressing a willingness to introduce it after 11 months of age [45].

Other barriers to the successful implementation of early food introduction recommendations are fear of a severe allergic reaction to the allergen on first consumption and lack of child cooperation. Caregivers need practical education on the safe and feasible introduction of peanuts into their child’s diet [46]. Some data have shown that older maternal age, non-Caucasian ethnicity, lower quality of life, identified feeding difficulties, and occurrence of allergic food reactions were associated with poorer compliance [29]. 

Guidelines of selected societies for peanut introduction into an infant’s diet are shown in Table 1. 

## 4. Immunotherapy

Immunotherapy is an alternative treatment for IgE-mediated FA, which is recommended by the EAACI. This method of treatment is particularly suitable for patients with a confirmed allergy who have not been introduced to peanuts early in their diet or who this method has not been successful for. Whereas various therapeutic strategies including sublingual and epicutaneous immunotherapy were evaluated, OIT is the most commonly applied strategy because of its efficacy and ease of use. OIT involves daily oral administration of the allergen to achieve desensitisation, understood as a temporary increase in threshold exposure to a food allergen. It typically consists of three parts: initial dose escalation, a build-up phase, and a maintenance phase. The initial dose is determined during oral food challenge (OFC) and is defined as the highest dose of allergen tolerated by the patient. The dose of allergen is increased every 1–2 weeks until the maintenance dose is achieved, according to a protocol designed by the investigators. Then, the maintenance dose (consistent quantity of the allergen) is taken daily for an extended period. This continuous exposure is intended to moderate the immune response to the allergen, causing desensitisation [14].

Data published in the literature (randomised clinical trials and uncontrolled clinical observations) demonstrate the efficacy of OIT in peanut allergy.

The PALISADE investigating group published an excellent randomised double-controlled trial of peanut immunotherapy. Participants (aged 4–55 years) received oral peanut-derived immunotherapy (300 mg peanut protein) for a 24-month maintenance phase. During the final food challenge, 67.2% of participants in the treatment arm of the study were able to consume ≥600 mg of peanut protein compared to 4% in the placebo group [47].

Jones and colleagues were the first to publish a randomised, placebo-controlled trial evaluating the effectiveness and safety of oral peanut immunotherapy in children aged 1–3 years. In this excellent study, the authors showed very high efficacy of OIT in the youngest children, with 71% of participants achieving desensitisation (defined as passing the 5000 mg peanut double-blind, placebo-controlled food challenge), compared with 2% of placebo-receiving children. The most significant finding from this study was achieving remission (a state of non-responsiveness after the discontinuation of immunotherapy) in 21% of immunotherapy-treated children [48]. Therefore, some authors have suggested the concept of salvage oral peanut immunotherapy in infants who do not benefit from early peanut introduction [49]. 

Greenhawt et al. conducted a phase 3 trial (DBPC) to assess the efficacy and safety of epidermal immunotherapy for peanut allergy. The study involved 362 participants aged 1–3 years. The efficacy of the applied therapy was evaluated after 12 months of using the epidermal patch (Vivaskin Peanut, maintenance dose 250 µg). The researchers showed that 67% of the study group achieved the primary endpoint compared to 33.5% of the placebo group. Severe adverse symptoms occurred in 8.6% and 2.5% of the groups, respectively [50]. 

A meta-analysis published in 2022 by Alghamdi and colleagues analysed the effectiveness of different methods of peanut immunotherapy. The most effective method of inducing desensitisation was OIT (74.3%). A total of 11% of patients who received sublingual immunotherapy (SLIT) achieved desensitisation compared to 61% of those who received subcutaneous (SCIT) and 49% of those who received epicutaneous (EPIT) immunotherapies [51]. This method represents a promising alternative, particularly for patients who cannot use oral immunotherapy due to intolerance.

There are numerous studies assessing the impact of peanut OIT on patients’ quality of life (QoL). Using standardised questionnaires, most authors found significant improvement in QoL, especially in emotional impact and a reduction in hypervigilance, social limitations, dietary restrictions, and dietary anxiety [52,53].

Although OIT seems to be effective in inducing desensitisation to peanut proteins, it also has some limitations. The main disadvantage is the occurrence of adverse events (AEs). Adverse reactions (generally mild) are reported in 50–95% of patients during OIT, especially in those with very severe allergy and/or long duration of a build-up phase [54]. The most frequent symptoms are mouth and throat itching and abdominal discomfort/pain. Unfortunately, 10–25% of individuals suffer from severe allergic reactions and require epinephrine injection [55,56]. According to a meta-analysis published by Grzeskowiak et al., AEs in peanut OIT led to the termination of treatment in 6.6% of children [57]. 

Researchers are continuously searching for additional treatment options for FA to improve the safety of OIT. To date, the monoclonal anti-IgE antibody omalizumab is the best-studied anti-IgE therapeutic. It is typically administered prior to the initiation of OIT to reduce AEs or symptoms associated with IgE-mediated allergic reactions during the build-up and maintenance phases. Clinical trials using omalizumab have shown promising results: reduction in the time required to achieve the maintenance dose, increase in cumulative tolerability to allergens, and increase in safety of OIT for single food allergens, such as peanuts [58,59]. Moreover, recent studies have determined the considerable potential of omalizumab—when used in conjunction with allergen avoidance—in reducing allergic reactions due to accidental exposure [60]. The duration of omalizumab treatment for IgE-FA is currently under investigation [61]. Another anti-IgE monoclonal antibody, currently subject to clinical trials for peanut allergy, is ligelizumab [62]. It is a next-generation humanised IgG1 mAb targeting IgE and has shown a number of possible advantages over omalizumab [63].

Recent data have demonstrated that ligelizumab has a 9-to-88-fold stronger suppression of free IgE with a 15-fold lower dissociation rate [64]. Ligelizumab has also been shown to have a 20-fold stronger inhibition in the binding of IgE to FceRI than omalizumab [63]. These results are extremely promising but are mainly from in vitro studies and will require further verification in future clinical trials. So far, studies of the role of ligelizumab in food allergy are limited (trial NCT04984876).

Dupilumab is an antibody directed against the common α subunit of the IL-4 and IL-13 receptors. According to the registration, it is used to treat asthma, AD, and nasal polyps. Studies are currently being conducted to investigate its efficacy in the treatment of peanut allergy in monotherapy and in combination with OIT (NCT03793608; NCT03682770). Of the other biologic drugs, etokimab (anti-IL-33) and abatacept (inhibits T-cell activation) remain in clinical trials. 

Recent food allergy therapies also include vaccines, nanoparticles, and Janus kinase and Bruton’s tyrosine inhibitors [65].

A recent meta-analysis published in *The Lancet* demonstrated that peanut OIT is associated with a higher risk of anaphylactic reactions requiring epinephrine than avoiding peanuts [66]. This is an unfavourable factor of ongoing treatment that should be discussed with patients and carers before the start of immunotherapy [67].

Patients should be provided with a personalised action plan to help them manage anaphylaxis [67]. It should be emphasised that patients treated with OIT take the allergen every day, at a dose many times higher than during accidental ingestion. Other limitations affecting the safety of OIT are cofactors associated with anaphylaxis such as infections, exercise, menstruation, temperature changes, and uncontrolled asthma [68,69]. 

Morris et al. reported a link between the ingestion of peanuts in the evening and the incidence of anaphylaxis resulting in the need to administer epinephrine. They analysed 307 patients undergoing immunotherapy, 31 (10%) of whom required the administration of epinephrine. Almost half (45.8%) of the reactions treated with epinephrine occurred in the evening, suggesting evening dosing as a possible contributing factor causing anaphylaxis in children undergoing peanut OIT [70]. 

Other disadvantages of oral immunotherapy include the need to take the allergen daily, frequent hospital visits, and the sheer length of the therapy [70]. 

It remains unclear whether OIT might contribute to the onset of eosinophilic esophagitis (EoE). Its occurrence is estimated to be between 0.5 and 5% in OIT [71]. The most common symptoms of EoE include food refusal, abdominal pain, dysphagia, recurrent nausea, and vomiting. However, diagnosis is complicated as gastrointestinal symptoms related to OIT are non-specific and subjective. The diagnosis of EoE has to be confirmed during digestive endoscopy with oesophageal biopsies. At this stage, longitudinal data of EoE are inadequate to determine the long-term safety of OIT [72,73]. 

**Figure 1:**
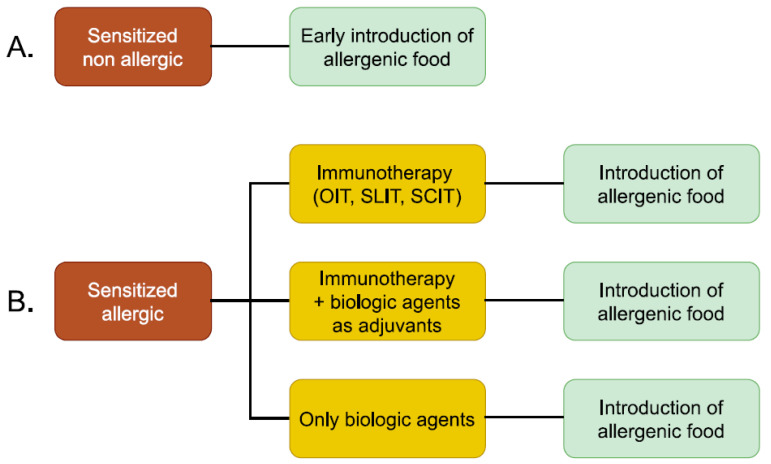
Potential treatment strategies for peanut allergy. (**A**) Treatment strategies for sensitized and non-allergic patients. (**B**) Treatment strategies for allergic patients.

Potential treatment strategies for peanut allergy are shown in Figure 1.

## 5. Conclusions

Until recently, the strategy of eliminating the allergen from the diet was the only treatment for peanut allergy. However, the outcomes of the LEAP and EAT studies revolutionised the approach to food allergy prevention, although only one-third of parents express a willingness to introduce peanuts into the diet of their children at an early age. In individuals whose peanut allergy has not been prevented, specific oral immunotherapy may be used. This method allows patients to develop desensitisation and, in some cases, tolerance to peanuts. Unfortunately, it is linked with frequent adverse reactions, including anaphylaxis. 

It seems that, in peanut allergy, prevention is better than the cure. However, the direct approach must be tailored to the individual patient and discussed in detail with the child and/or their parents.

## Figures and Tables

**Table 1:** Guidelines of selected societies for peanut introduction into diet.

Society	Breastfeeding Exclusively	Peanut Introduction
American Academy of Allergy, Asthma and Immunology (AAAAI) (2020) [39]	Initiate in the first 4–6 months of life	At around 6 months, but not before 4 months
British Society for Allergy & Clinical Immunology (BSACI) (2019) [37]	Initiate in the first 4–6 months of life	At 4 months in children at high risk of developing FA
European Academy of Allergy and Clinical Immunology (EAACI) (2020) [38]	Initiate in the first hour after birth and exclusively breastfeed for 6 months of life	At 4–6 months
Australasian Society of Clinical Immunology and Allergy (2020) [41]	Exclusive breastfeeding for the first 6 months of life	At around 6 months
Canadian Paediatric Society (CPS) (2021) [43]	Breastfeeding for the first 6 months of life	At around 6 months, but not before 4 months of age—for a high-risk infant In infants at low risk—around 6 months of age

## Abbreviations

AEs	adverse events
CPS	Canadian Paediatric Society
EAACI	European Academy of Allergy and Clinical Immunology
EoE	eosinophilic esophagitis
EPIT	epicutaneous immunotherapy
EU	European Union
FA	food allergy
FS	food sensitisation
sIgE	specific immunoglobulin E
OIT	oral immunotherapy
PAL	precautionary allergen labelling
QoL	quality of life
SCIT	subcutaneous immunotherapy
SLIT	sublingual immunotherapy
SPT	skin prick test
WHO	World Health Organisation
